# Expanding Indications for Epidural Spinal Cord Stimulation in Spinal Cord Injury

**DOI:** 10.3390/jcm15207936

**Published:** 2026-10-14

**Authors:** Ron Gadot, Yi Lu

**Affiliations:** Department of Neurosurgery, Brigham and Women’s Hospital, Boston, MA 02115, USA; rgadot@mgb.org

**Keywords:** spinal cord stimulation, epidural stimulation, spinal cord injury, neuromodulation, autonomic dysreflexia, motor recovery, neurorehabilitation, clinical trials, post-stroke hemiparesis

## Abstract

**Background/Objectives**: Epidural spinal cord stimulation (ESCS) has long been used to treat chronic neuropathic pain, but a growing body of evidence supports its use as a neuromodulatory intervention for spinal cord injury (SCI) itself, targeting motor, autonomic, bladder, bowel, and respiratory deficits rather than pain. Landmark studies show that lumbosacral and cervical ESCS, combined with activity-based rehabilitation, can enable volitional movement, standing, and stepping after chronic, motor-complete SCI, with gains persisting after stimulation is switched off in a subset of participants. For cardiovascular indications, recent head-to-head evidence indicates that the last three thoracic spinal cord segments, rather than the lumbosacral cord used in earlier studies, are the optimal stimulation target for safe, effective blood pressure regulation. **Methods**: Narrative Review. **Results**: This review summarizes the mechanistic rationale for ESCS in SCI, synthesizes recent (2022–2026) clinical and safety data across motor, cardiovascular, and bladder/bowel domains, catalogs the current international landscape of ongoing clinical trials grouped by level of evidence, and reports perspectives from the SCI patient community and treating clinicians on the priorities, barriers, and ethical considerations that will shape clinical translation. We also briefly contextualize ESCS for SCI within two non-SCI applications most mechanistically and translationally proximate to this literature—post-stroke upper-limb hemiparesis and cardiac autonomic modulation. **Conclusions**: We conclude with a discussion of methodological and translational barriers—small sample sizes, limited blinding, procedural risk, and variable responder profiles—that must be addressed before ESCS for SCI can move from investigational feasibility studies toward standard-of-care therapy.

## 1. Introduction

Spinal cord stimulation was first introduced in 1967 as a treatment for intractable pain, based on Melzack and Wall’s gate-control theory, and its approved indications have since expanded to failed back surgery syndrome, complex regional pain syndrome, painful diabetic peripheral neuropathy, and refractory angina [1,2]. In these traditional applications, electrodes are placed in the dorsal epidural space—typically over the thoracic or cervical spinal cord—to activate large-diameter dorsal column afferents and engage descending inhibitory pathways that dampen nociceptive transmission [2].

Over the last decade and a half, a parallel and largely independent line of investigation has repurposed the same anatomical target—the dorsal epidural space—for a fundamentally different therapeutic goal: enabling and, in select cases, durably improving function after spinal cord injury. This “nontraditional” application does not primarily target pain; a 2025 pilot study reported concurrent reductions in neuropathic pain alongside motor and autonomic gains during epidural stimulation in individuals with sensorimotor-complete SCI, suggesting the distinction from pain-focused ESCS is one of primary intent rather than an absolute mechanistic separation [3]. The therapeutic rationale instead rests on the observation that even in clinically “motor-complete” SCI, a small number of spared descending axons frequently cross the lesion [4,5]. Tonic or spatiotemporally patterned electrical stimulation delivered over the lumbosacral or cervical enlargement raises the excitability of spinal interneuronal networks below the injury to a level at which these sparse residual descending signals—augmented by proprioceptive afferent feedback—become sufficient to drive coordinated, weight-bearing movement, or to regulate autonomic outflow that is otherwise disconnected from supraspinal control [4,5,6,7].

This review focuses specifically on epidural (surgically implanted) spinal cord stimulation for spinal cord injury, as distinguished from transcutaneous (skin-surface) spinal stimulation, which uses a related but mechanistically distinct, noninvasive approach and has its own separate evidence base (including the FDA-cleared ARC-EX system for upper-limb function) [8,9]. We summarize recent clinical evidence (concentrating on 2022–2026 publications) across three nontraditional domains: (1) motor recovery of the lower and upper limbs, trunk, and sensory function, (2) autonomic recovery, including blood pressure regulation and bladder/bowel/sexual function, and (3) emerging applications such as respiratory function. A 2024 systematic review pooling 64 studies and 306 patients confirmed that reported benefits of epidural stimulation for chronic SCI extend beyond locomotor recovery to cardiovascular, respiratory, and genitourinary domains, providing a quantitative anchor for the qualitative synthesis presented here [10]. Because ESCS is fundamentally a platform technology for spinal neuromodulation, we additionally augment this SCI-focused evidence within two non-SCI applications most mechanistically and translationally proximate to it: post-stroke hemiparesis, which uses the identical cervical afferent-recruitment mechanism as SCI upper-limb recovery, and cardiac autonomic modulation, which extends the same thoracic sympathetic-circuit rationale as SCI cardiovascular regulation. Finally, we report perspectives from the SCI patient community and treating clinicians on the priorities and barriers that will shape clinical translation of this work.

### Search Strategy

This narrative review draws on literature and trial registry records identified through PubMed/MEDLINE, ClinicalTrials.gov, the WHO International Clinical Trials Registry Platform (ICTRP), and the Chinese Clinical Trial Registry (ChiCTR), searched through July 2026, with the registry records for the trials in Table 1 updated in October 2026. Search terms combined “epidural spinal cord stimulation” or “epidural electrical stimulation” with “spinal cord injury” and indication-specific terms (e.g., “blood pressure”, “autonomic dysreflexia”, “bladder”, “walking”, “upper limb”, “respiration”). The primary focus was English-language, peer-reviewed publications from 2022 to 2026, supplemented by foundational studies published earlier where necessary for mechanistic or historical context; reference lists of identified reviews were hand-searched for additional citations. Case reports, case series, feasibility-study protocols, and registered but not-yet-published trials were included given the early-phase nature of this field and are identified as such in the text. Non-peer-reviewed sources (institutional or press websites) were used sparingly, only where no peer-reviewed alternative existed for a specific factual claim such as trial recruitment status. This approach follows recommended practice for narrative reviews, which are not required to conform to PRISMA reporting standards [11].

To address potential overlap between registries, ClinicalTrials.gov and ChiCTR records were cross-referenced by sponsor, site, and study title; no trial was identified as dually registered under both systems, and the two ChiCTR-only entries in Table 1 (the EMG-induced rehabilitation trial and the observational functional-recovery study) are listed separately because no corresponding ClinicalTrials.gov record could be located for either. For terminology used in Table 1 and the text: a study is classified as “recruiting” if the registry record’s recruitment-status field was so listed at the time of the October 2026 registry update; “ongoing” denotes any non-terminal status other than recruiting or completed (e.g., active-not-recruiting, enrolling by invitation); “not yet recruiting,” “completed,” and “unknown” (registry status not verified for more than two years) are reported as listed in the registry record; and “recently registered” or “recently initiated” denote a first-posted or registration date within the 24 months preceding the search (July 2026). Beyond the search terms listed above, records were additionally screened for eligibility by confirming that the intervention involved an implanted (epidural)—rather than transcutaneous or intraspinal—stimulation device, and that spinal cord injury was a primary rather than incidental study population; records failing either criterion were excluded from Table 1.

## 2. Mechanistic Rationale

The rationale for ESCS in SCI differs fundamentally from its use in chronic pain. In SCI, epidural electrodes placed over the dorsal roots of the lumbosacral (for lower-limb and pelvic-organ function) or cervical (for upper-limb function) enlargements preferentially recruit large-diameter proprioceptive (group Ia/II) afferent fibers as they enter the dorsal horn [4,39]. This afferent volley raises the membrane potential of spinal motor and premotor interneuronal circuits closer to firing threshold—a state sometimes described as converting an otherwise “silent” or subthreshold descending command into a suprathreshold, functionally effective one [5,39]. Because the effect depends on convergence between electrically evoked afferent input and any surviving supraspinal drive, stimulation-enabled movement is typically volitional (patient-initiated) rather than a fixed, stimulus-locked reflex, and is often lost within seconds of the stimulator being switched off—an important and consistent limitation across studies to date [6,40].

Two complementary stimulation paradigms have been described. Continuous (“tonic”) stimulation at fixed parameters can enable standing and some stepping-like activity, particularly when combined with intensive locomotor training [6]. Spatiotemporal or “activity-dependent” stimulation—in which stimulation location and timing are dynamically adjusted across an electrode array to mimic the natural sequence of motor pool activation during a movement cycle—has produced more robust and rapid restoration of walking, including in individuals with chronic, complete paralysis [7,40,41]. Closed-loop and brain-controlled variants, in which stimulation is triggered or modulated by cortical signals from an implanted brain–computer interface (BCI), represent the most recent evolution of this approach and are discussed in Section 5 [42,43].

For autonomic applications, lumbosacral or lower-thoracic ESCS is thought to engage sympathetic preganglionic circuitry and spinal reflex arcs that regulate vasomotor tone, enabling graded elevation of blood pressure in individuals with injury-induced hypotension, and modulation of detrusor–sphincter coordination for bladder and bowel control. A 2025 study substantially advanced this mechanistic picture by mapping the neuronal architecture that drives pathological autonomic dysreflexia after SCI and showing that it is overlapping with, but distinguishable from, a competing circuit that epidural stimulation can engage to safely regulate blood pressure, with both converging on a shared spinal interneuron subpopulation; this provides a mechanistic explanation for how the same general stimulation target can both suppress dysreflexic hypertension and support adaptive blood pressure regulation (see Section 4.1) [44].

Regarding anatomical terminology used throughout this review, we distinguish three related but frequently conflated concepts. The spinal cord (neurological) segment is defined by its corresponding dorsal/ventral root exit level and may be discordant with the adjacent vertebral column in adults, because the cord itself terminates near the L1–L2 vertebral level (the conus medullaris); the vertebral level is the bony landmark used for surgical entry (e.g., a laminectomy performed at a given thoracic or lumbar vertebral level to access the corresponding, more caudally displaced, spinal cord segments); and the electrode contact or stimulation level refers to the specific contacts on an implanted array that are active during a given program, which can be reprogrammed across several spinal cord segments without any change in the electrode’s fixed physical (vertebral) position. Where the primary literature specifies segmental targets—for example, the lower thoracic spinal cord segments used for cardiovascular regulation (Section 4.1)—we report the neurological segment; where only the surgical vertebral level is specified in the source material, we report it as such and note the distinction explicitly rather than treating the two as interchangeable.

The heterogeneity of ESCS effects across the applications reviewed in this article—robust, reproducible motor gains with lumbosacral and cervical stimulation for SCI (Section 3); consistent but mechanistically distinct cardiovascular regulation via thoracic sympathetic circuitry (Section 4.1); and more variable outcomes in non-SCI, non-neurological applications such as heart failure (Section 10.2)—likely reflects differences in the underlying circuitry engaged rather than a single unified mechanism of action. Motor and trunk applications depend on convergence between electrically evoked large-diameter afferent input and surviving supraspinal descending drive within intact segmental interneuronal networks, and accordingly require substantial residual neural substrate below the injury, such that benefit should track the extent of spared white matter and network integrity. Cardiovascular and pelvic-organ applications instead engage largely autonomic (sympathetic preganglionic and visceral afferent) circuitry that can remain functionally stimulable even after clinically complete SCI, which may explain why autonomic benefit is reported even in cohorts selected for absent volitional motor function. By contrast, applications outside the spinal cord’s usual afferent-motor or autonomic circuitry altogether—such as cardiac sympathetic modulation in heart failure—rely on more indirect mechanisms, plausibly accounting for their comparatively inconsistent evidence base (Section 10). This circuit-based framework is offered as a working hypothesis, rather than an established unifying mechanism, to explain why ESCS produces reliable benefit in some domains and null or preliminary results in others, and it suggests that future mechanistic studies should be organized around the specific neural circuit engaged by a given stimulation target rather than around ESCS as a monolithic intervention.

## 3. Motor Recovery

### 3.1. Lower-Limb Standing, Stepping, and Walking

The foundational human evidence for ESCS-enabled motor recovery after motor-complete SCI came from the University of Louisville/UCLA collaboration, which first demonstrated stimulation-enabled standing and, subsequently, voluntary movement of the legs in four individuals with chronic, clinically motor-complete SCI [45]. The Mayo Clinic group extended this work, reporting that a young man with a T6 motor-complete injury regained the ability to stand and take steps with body-weight support while epidural stimulation was active, though function was lost when the device was turned off [40].

The most substantial recent advances have come from the Lausanne group (EPFL/CHUV), led by Grégoire Courtine and colleagues. In 2018, this group described a “targeted neurotechnology” approach using spatiotemporal epidural stimulation, enabling voluntary, adaptable walking, cycling, and swimming in individuals with incomplete SCI [41]. A 2022 study in Nature Medicine extended this to three individuals with chronic, complete sensorimotor paralysis, in whom activity-specific spatiotemporal stimulation enabled standing, walking, cycling, swimming, and trunk control within a single day [7]. A companion study in Nature reported that spatiotemporal stimulation combined with intensive neurorehabilitation (“EESREHAB”) restored walking in nine individuals with chronic SCI, with walking recovered even in the absence of stimulation in four participants, and identified a specific population of spinal interneurons whose activation is necessary for stimulation-enabled walking recovery [46]. In 2023, the same group reported a “brain–spine interface” (BSI)—pairing implanted cortical electrodes with spatiotemporal epidural stimulation of the lumbar cord—that enabled more naturalistic, intention-driven walking and ambulation over complex terrain in a participant with chronic SCI; as with most stimulation-enabled (rather than EESREHAB-style stimulation-independent) walking described in this section, this benefit was present while the device was active rather than shown to persist off-stimulation [42]. A 2025 report in Science Translational Medicine further showed that high-frequency epidural stimulation can reduce spasticity while simultaneously facilitating walking recovery, addressing a long-standing concern that stimulation parameters optimized for movement might exacerbate spasticity [47].

Smaller case studies and case series published in 2025 continue to refine patient selection and stimulation protocols: a case study of a T11–T12 injury involving the conus medullaris demonstrated that individualized mapping of stimulation parameters could improve isolated hip flexor, trunk, and abdominal muscle activation sufficient to support walking with a walker [48], and a case series combining epidural stimulation with robotic gait training, motor imagery, and trunk rehabilitation reported synergistic functional gains in chronic, sensorimotor-complete SCI [49]. A 2025 study in the Journal of NeuroEngineering and Rehabilitation reported that activity-based recovery training combined with cervical epidural stimulation improved standing performance in individuals with cervical SCI, extending stimulation-assisted standing paradigms beyond thoracic and lumbar injury levels [50].

### 3.2. Upper-Limb and Hand Function

Restoring hand and arm function is a high priority for individuals with cervical SCI, and cervical ESCS has emerged as a distinct research focus. A 2025 narrative review summarized the rationale and early clinical evidence for cervical spinal cord stimulation in upper-limb paralysis, concluding that stimulation over the cervical enlargement can enhance volitional grip strength and reaching in tetraplegia through mechanisms analogous to those described in the lumbar cord [51]. A 2025 first-in-human study combined an implanted brain–computer interface with cervical epidural stimulation (BCI-ESCS) in a participant with chronic, motor-complete cervical SCI; this closed-loop approach potentiated corticospinal connectivity, producing both immediate improvements in grip strength and object manipulation and increased corticospinal excitability after a single session, exceeding gains seen with tonic (non-BCI-triggered) stimulation alone. This report is currently a preprint pending peer review [43].

It is worth noting, for context, that the largest published trial of spinal stimulation for upper-limb function in tetraplegia to date—the Up-LIFT trial, published by Moritz and colleagues in Nature Medicine in 2024—enrolled 65 participants, of whom 60 completed the study protocol; 72% of completers were classified as responders, meeting pre-specified improvement criteria in both strength and functional domains. The trial used a noninvasive, transcutaneous system (ARC-EX), not an implanted epidural device; it is included here only to situate the implanted (ARC-IM) work within the broader device landscape, since ARC-EX’s commercial and regulatory success (FDA clearance) has substantially accelerated investment in the implanted successor systems discussed below [8,9].

### 3.3. Trunk Control and Postural Stability

Beyond limb movement, epidural stimulation of the lumbosacral cord has been shown to improve seated trunk stability and reaching in individuals with severe thoracic SCI, suggesting that stimulation-enabled improvements extend to postural and core stabilization circuits that are essential for functional independence in wheelchair users, not solely to overground ambulation [52].

### 3.4. Sensory Restoration

Motor-focused sublesional stimulation does not, by itself, restore impaired sensation below the injury. A 2026 report from a Brown University group in Nature Biomedical Engineering described “perilesional” stimulation—delivered above as well as below the lesion—that evoked lower-limb sensations synchronized with ongoing movement in three participants with chronic, motor-complete SCI, enabling accurate reporting of leg position and representing an early but notable step toward combined sensorimotor (rather than purely motor) restoration with epidural stimulation [53].

## 4. Autonomic Recovery

### 4.1. Cardiovascular Regulation

Cardiovascular dysautonomia—including chronic hypotension, orthostatic intolerance, and autonomic dysreflexia—affects a large proportion of individuals with cervical and high-thoracic SCI and is consistently ranked by patients as a higher priority for treatment than ambulation [54]. Foundational work from the Louisville/Kentucky group demonstrated that individualized lumbosacral (L1–S1) epidural stimulation programs could normalize blood pressure from a chronic hypotensive baseline into a stable, functional range in individuals with severe cervical and upper-thoracic SCI, an effect that in some participants persisted for months [54]. A related early feasibility study in four individuals with SCI-associated orthostatic hypotension and autonomic dysreflexia found that stimulation over spinal segments L1–S1 could reliably raise systolic blood pressure into a target range of 105–120 mmHg [54]. Follow-up work demonstrated sustained, trainable improvements in cardiovascular function with repeated stimulation sessions in individuals with chronic cervical SCI [55].

A major recent update refines this picture of the optimal stimulation target for cardiovascular indications. While the foundational studies above used lumbosacral (L1–S1) stimulation, Phillips and colleagues reported in 2025 a head-to-head, within-participant comparison (n = 14) demonstrating that epidural stimulation must instead target the last three thoracic spinal cord segments (neurological segments, not vertebral levels; given the discordance between cord and column discussed in Section 2). The corresponding electrode array is typically implanted at a more rostral thoracic vertebral level—rather than the lumbosacral cord—to achieve safe and effective blood pressure regulation; lumbosacral stimulation, by contrast, produced less reliable and, in some cases, inadequate hemodynamic responses in the same participants [56]. This clinical finding builds directly on earlier translational work by Squair and colleagues, who developed a closed-loop “neuroprosthetic baroreflex”—biomimetic epidural stimulation titrated in real time to beat-to-beat blood pressure—that controlled hemodynamics in rodents, non-human primates, and humans after both acute and chronic SCI, and first identified the lower thoracic cord as the physiologically appropriate target for this approach [57]. Mechanistically, the thoracic-targeting strategy is further supported by the dissociable-but-overlapping autonomic circuitry described above, in which epidural stimulation of thoracic sympathetic outflow engages a spinal interneuron population distinct from—but convergent with—the one driving autonomic dysreflexia [44]. Together, these findings have direct implications for the design of ongoing and future cardiovascular ESCS trials (Section 6; Table 1), several of which were originally designed around lumbosacral targets and may need to incorporate or transition to thoracic stimulation protocols as this evidence matures.

The Epidural Stimulation After Neurologic Damage (E-STAND) trial, designated phase II in its published protocol and based at the University of Minnesota, was designed explicitly to evaluate both volitional movement and cardiovascular outcomes in individuals with chronic, motor-complete SCI, including a dedicated subgroup with cardiovascular dysautonomia for blood-pressure-stabilization optimization [58,59]. Investigators have reported that E-STAND has demonstrated stimulation-enabled recovery of some volitional movement alongside improved cardiovascular, bowel, and bladder function, and describe it as the second-largest study to date evaluating epidural stimulation for thoracic SCI [59]. A systematic review published online in June 2026 synthesized the growing evidence base on spinal cord stimulation for autonomic dysfunction after SCI, reinforcing cardiovascular regulation as one of the most consistently reproducible nontraditional benefits of ESCS across independent research groups [60]; a complementary 2026 scoping review reached similar conclusions across cardiovascular, bowel, lower urinary tract, and sexual domains [61].

ONWARD Medical’s implanted ARC-IM platform—distinct from its noninvasive, FDA-cleared ARC-EX system—has been used in human feasibility studies since 2022 to evaluate hemodynamic stabilization and trunk control alongside mobility outcomes, reflecting industry convergence toward multi-indication implanted stimulation platforms [9,31].

### 4.2. Bladder, Bowel, and Sexual Function

Neurogenic bladder and bowel dysfunction remain among the most burdensome consequences of SCI, and epidural stimulation research in this domain builds on an NIH-supported consortium framework established in the mid-2010s to formally structure clinical research on bladder, bowel, and sexual function outcomes [62]. Subsequent case-level work has shown that lumbosacral epidural stimulation can acutely modulate lower urinary tract and bowel function [63], improve coordination between the detrusor muscle and external urethral sphincter, and—in a four-month bladder-mapping study—identify stimulation frequencies (approximately 30 Hz in one reported case) that minimized post-void residual volume [64]. A larger 2022 study performed functional bladder mapping during filling cystometry in seven individuals with motor-complete SCI and identified specific stimulation parameters that improved bladder compliance while maintaining stable blood pressure and enabled initiation of voiding, providing a more systematic parameter-mapping framework than earlier single-case reports [65]. A related registered study (SPARC) continues systematic bladder mapping and training in this population [37]. Beyond voiding, a 2023 case series in three individuals with cervical or upper-thoracic motor-complete SCI—published in this journal—showed that real-time lumbosacral epidural stimulation applied during digital anorectal stimulation, a common trigger of autonomic dysreflexia during bowel care, kept systolic blood pressure elevations below the diagnostic threshold for autonomic dysreflexia in all participants, providing a concrete, mechanism-linked demonstration of AD prevention rather than a general impression of “fewer episodes” [66].

### 4.3. Respiratory Function

An emerging and comparatively less mature application involves the effect of cervical and high-thoracic epidural stimulation on respiratory drive and diaphragmatic function after SCI. This remains at an early, largely mechanistic stage relative to motor and cardiovascular applications but is now the subject of active clinical investigation (Section 6) [38].

## 5. Evolution of Stimulation Approaches

Three technological trends are shaping the next generation of ESCS for SCI. First, spatiotemporal, multi-electrode arrays that dynamically pattern stimulation across the cord—rather than delivering fixed, continuous stimulation—have produced the most robust motor outcomes reported to date and are increasingly standard in leading academic programs [7,40,41]. Second, closed-loop and brain-controlled systems, which trigger or modulate stimulation based on cortical signals or physiological feedback (e.g., real-time blood pressure), are moving from single-case proofs of concept toward small controlled feasibility trials, and represent a shift from open-loop “assistive” stimulation toward systems designed to also drive activity-dependent neuroplasticity; the closed-loop “neuroprosthetic baroreflex” for blood pressure control (Section 4.1) is an example of this trend applied to an autonomic rather than motor endpoint [42,43,57]. Third, there is a growing bifurcation—and complementary relationship—between noninvasive transcutaneous systems, which offer lower procedural risk and are more amenable to early-phase and pediatric investigation, and implanted epidural systems, which offer greater spatial selectivity and are generally required for the more complex spatiotemporal and closed-loop paradigms described above; several device developers are now pursuing parallel noninvasive and implanted product lines for related indications [8,9].

## 6. Ongoing Clinical Trials

**Figure 1:**
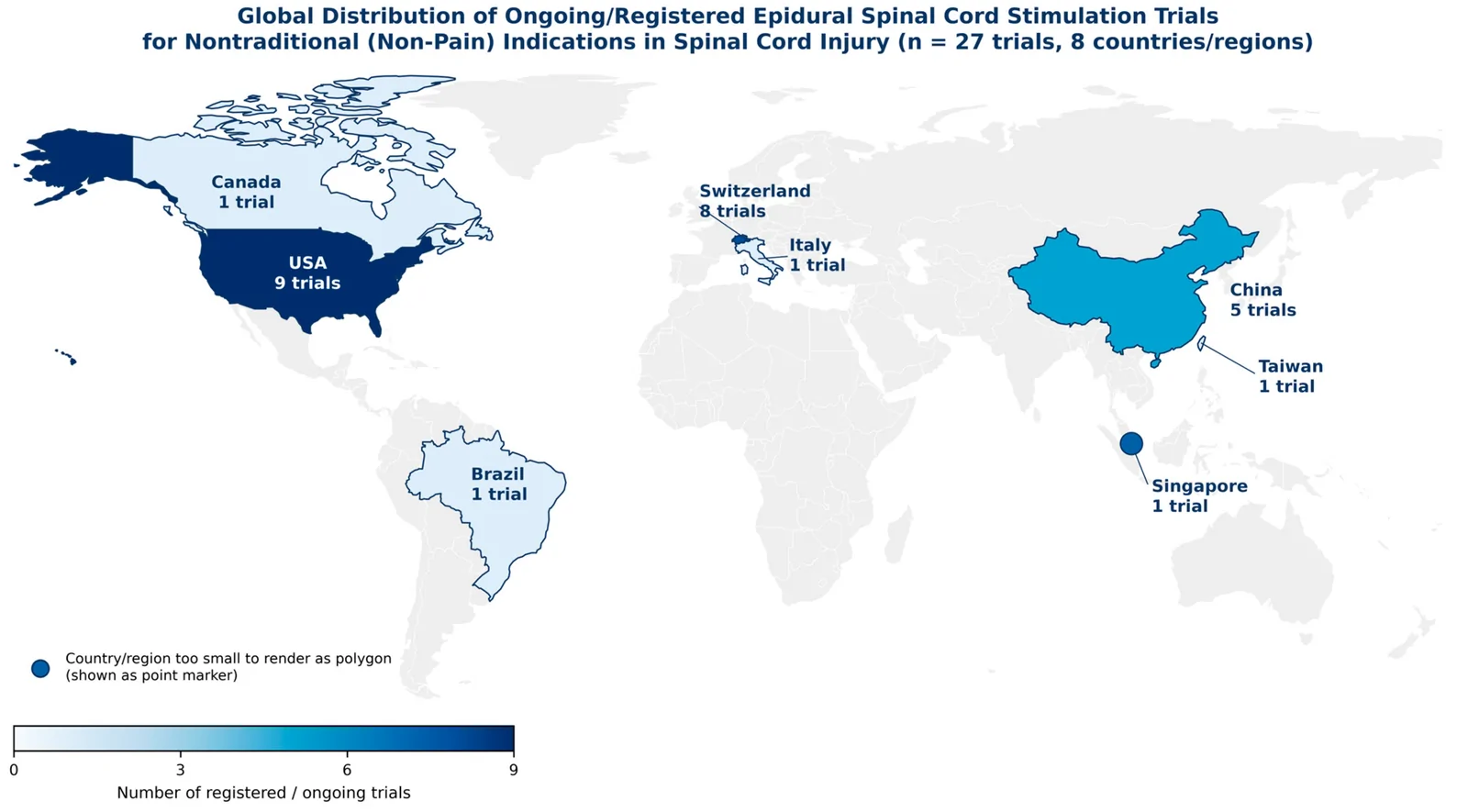
Global distribution of ongoing or recently registered epidural spinal cord stimulation trials for nontraditional (non-pain) indications in spinal cord injury, by country/region (n = 27 trials across 8 countries/regions, as of July 2026). Color intensity reflects the number of trials per country/region (choropleth, Natural Earth 1:110 m boundaries); Singapore is plotted as a point marker because its land area is not resolvable as a polygon at this map resolution. Counts correspond to the trials listed in Table 1. Image generated with the assistance of Claude Sonnet (Anthropic) modeling.

Table 1 summarizes trials evaluating epidural spinal cord stimulation for nontraditional (non-pain) indications in spinal cord injury, drawn from ClinicalTrials.gov and cross-checked against the WHO ICTRP network and the Chinese Clinical Trial Registry (ChiCTR), grouped by primary therapeutic domain (motor/mobility, cardiovascular/autonomic, bladder/bowel/sexual, and respiratory) to make the relative maturity and size of the pipeline accessible at a glance. Phase/design classification and target enrollment (N) are included where reported in the registry record; “N/A” indicates that no formal phase is assigned in the registry record—many early-phase device studies are not assigned a formal FDA phase and are recorded as such in their registry entries. The pipeline is international: in addition to numerous United States sites, active or recently completed programs are based at the Centre Hospitalier Universitaire Vaudois/École Polytechnique Fédérale de Lausanne (CHUV/EPFL) in Switzerland, the National Neuroscience Institute in Singapore, academic centers in China (Zhejiang University, registered on ClinicalTrials.gov, and separate ChiCTR-registered studies not cross-listed on ClinicalTrials.gov), IRCCS Ospedale San Raffaele in Milan, Italy, Buddhist Tzu Chi General Hospital in Taiwan, the University of Calgary and the University of British Columbia in Canada, and the University of São Paulo General Hospital in Brazil. Figure 1 summarizes the geographic distribution of the 27 trials identified across these eight countries/regions; the full trial-level detail, grouped by domain, is provided in Table 1. This list is illustrative of the current trial landscape rather than exhaustive, and study status should be independently verified given the pace of registration in this field.

## 7. Challenges and Limitations

Despite consistent and increasingly reproducible signal across independent centers, several barriers stand between current evidence and routine clinical adoption. Most published series remain small (single-digit to low double-digit cohorts), reflecting the intensive resource requirements of surgical implantation, individualized electrode-array mapping, and months of supervised activity-based training; this limits statistical power and generalizability [7,59,67].

Safety data specific to this population are reassuring but still limited. A dedicated safety analysis of the first 14 E-STAND participants found no surgical complications and only one device malfunction (occurring four months post-implantation), with no adverse events reported despite stimulation applied for up to 23 h per day across a broad parameter range (frequency 18–700 Hz, pulse width 100–600 µs, amplitude 0.4–17 mA) [68]. However, cardiovascular monitoring during stimulation-parameter optimization is warranted: a separate analysis found that autonomic dysreflexia occurred in 22% of parameter-testing sessions conducted during motor-focused epidural stimulation, though 97% of these episodes were asymptomatic and self-limited—underscoring that overall safety is favorable, but that off-target cardiovascular effects during mapping are not negligible [69].

True blinding and sham-controlled designs are difficult to implement in implanted (epidural) studies, since stimulation-enabled movement is often immediately and subjectively apparent to both participant and investigator, raising the risk of expectation and placebo effects influencing outcome assessment [7]. Notably, the noninvasive transcutaneous stimulation literature has already produced a multicenter, triple-blind, sham-controlled randomized trial (eWALK), which found that 12 weeks of transcutaneous stimulation combined with locomotor training provided no additional improvement in walking ability over locomotor training alone [70]. This sobering result both illustrates a design standard that implanted ESCS research has not yet matched and raises the possibility that some of the benefit attributed to stimulation in open-label implanted studies could reflect training, expectation, or natural variability rather than the stimulation itself—reinforcing the need for controlled designs as implanted-device trials mature.

Response is heterogeneous: benefit appears to depend on the extent of spared descending white matter, injury level and completeness, and time since injury, and reliable pre-implantation biomarkers to identify likely responders are still under development (e.g., the imaging-biomarker approach in NCT06438991) [18,67]. Device-related risks—infection, lead migration, and the need for surgical revision—are nontrivial in a population already at elevated risk for skin and wound complications. Finally, most reported motor benefits remain stimulation-dependent (lost when the device is switched off), meaning the intervention should currently be framed as an assistive/enabling technology for most patients rather than a cure, with the 2022 EESREHAB report representing a notable, but still singular, demonstration of persistent recovery after prolonged combined stimulation and rehabilitation [40,46].

Because study designs across the domains and applications reviewed in this article range from single case reports to randomized controlled trials, Table 2 summarizes the highest level of clinical evidence currently available for each major application discussed, to make these differences explicit rather than implicit in the narrative text above.

## 8. Patient and Clinician Perspectives

Whether ESCS for SCI ultimately reaches clinical practice depends not only on efficacy and safety data but on whether the therapy addresses outcomes that people with SCI themselves prioritize, and on whether treating clinicians perceive the barriers to implementation as surmountable. A 2023 survey-based study of 223 members of the SCI community—the large majority with traumatic SCI and substantial representation across injury levels and completeness—found that recovery of bladder, bowel, and sexual function and trunk/hand function were rated among the highest-priority outcomes, alongside more commonly discussed goals such as walking; respondents were broadly willing (91%) to commit to a structured post-implantation training/rehabilitation protocol, and most expressed willingness to accept surgical risk in exchange for meaningful functional gains, though risk tolerance varied with the specific benefit offered and its durability [73]. These findings reinforce a theme raised implicitly throughout this review: bladder, bowel, sexual, and autonomic outcomes (Section 4), while less visible in the media than walking recovery, are of comparable or greater importance to the people who would receive this therapy, and trial design and outcome selection should reflect that prioritization rather than defaulting to ambulation as the primary endpoint.

A complementary survey of SCI medicine physicians at United States SCI Model Systems sites (conducted in January 2019; 42 respondents) identified the principal barriers clinicians perceive to clinical implementation of epidural stimulation for functional improvement: the need for additional efficacy studies (92.9%), the lack of clear guidelines on stimulation parameters (83.3%), uncertainty about which patients are most likely to benefit (76.2%), the lack of targeted post-operative training protocols (69.1%), limited availability of surgical sites (69.1%), and the monetary cost of the device and its implantation (54.8%) [74]. The same analysis framed these barriers around the ethical construct of equipoise and noted that physicians most often reported that patients learned of epidural stimulation from news media stories (84.6%) [74]. Several ethical considerations are directly relevant to the trials summarized in Table 1: appropriate informed consent given the still-investigational and stimulation-dependent nature of most reported benefit (Section 7); equitable access, given that current implementation is concentrated in a small number of academic centers with specialized rehabilitation infrastructure, creating a risk that early clinical translation of ESCS could disproportionately benefit patients with geographic or socioeconomic access to these centers; and management of patient expectations in a therapeutic area that has received substantial lay-press attention, which can outpace the actual evidentiary maturity described in Section 3, Section 4, Section 5, Section 6 and Section 7 above. Taken together, the patient-priority data above and these clinician-identified barriers suggest that successful translation of ESCS for SCI, beyond further efficacy trials, will require parallel investment in reimbursement pathways, standardized training infrastructure, and clear, expectation-calibrated patient communication.

## 9. Future Directions

Several trends are likely to shape the field over the next several years. Closed-loop, BCI-driven stimulation is likely to expand beyond single-case demonstrations into small controlled trials, particularly for upper-limb applications where corticospinal signal quality is more favorable [42,43]. Personalized, imaging- or electrophysiology-based biomarkers for responder selection are an active area of investigation and could substantially improve trial efficiency and patient counseling [18]. Combination approaches—pairing ESCS with pharmacological agents, robotic rehabilitation, or transcutaneous “priming” stimulation—are being explored as a means of accelerating or deepening functional gains [49]. Extension to subacute (rather than only chronic) SCI populations, where residual plasticity may be greater, and to pediatric SCI, are natural next steps once safety and efficacy are further established in adults. Cardiovascular trial design in particular will need to incorporate the thoracic-targeting evidence discussed in Section 4.1, and closed-loop, biomarker-guided stimulation such as the neuroprosthetic baroreflex approach may become the standard for autonomic indications specifically [56,57]. Finally, as implanted platforms such as ARC-IM mature alongside their noninvasive counterparts, the field will need consensus outcome measures and, eventually, randomized controlled or stepped-wedge trial designs—of the kind already demonstrated to be feasible by eWALK—to support regulatory approval and reimbursement for indications beyond pain [70].

## 10. ESCS Beyond Spinal Cord Injury: Translationally Relevant Non-SCI Applications

The mechanistic principles developed above—unmasking residual descending motor drive via afferent recruitment (Section 2), and modulating sympathetic vasomotor and visceral outflow (Section 4)—are not unique to SCI. Several other non-SCI applications including critical limb ischemia and peripheral vascular disease [75,76,77,78,79,80], painful diabetic peripheral neuropathy [81,82,83,84], gastrointestinal motility disorders [85], asthma [86], and disorders of consciousness [87,88,89,90,91]—are longstanding or investigational uses of spinal cord stimulation with their own separate literatures, but are more mechanistically distant from the SCI-restorative paradigm that is this article’s focus and are accordingly out of scope here; interested readers are directed to the vascular-, neuropathic-pain-, and disorders-of-consciousness-focused reviews cited above for a fuller treatment of those indications.

### 10.1. Post-Stroke Upper-Limb Hemiparesis

The closest non-SCI parallel to the motor work described in Section 3 comes from post-stroke hemiparesis. Cerebral stroke, like SCI, can disrupt descending corticospinal commands while leaving downstream spinal motor circuits largely intact, providing the same rationale for afferent-recruitment stimulation used in SCI. In a first-in-human study, Powell and colleagues implanted two epidural leads over the cervical spinal roots (C3–T1) in two participants with chronic, moderate-to-severe post-stroke arm and hand weakness; continuous stimulation improved grip force by 40% and 108% in the two participants, improved movement speed by 30–40%, and enabled functional tasks (such as independently eating with utensils) that had not been possible for years, with some gains persisting even after stimulation was turned off and no serious adverse events reported [92]. A subsequent feasibility trial by the same group (University of Pittsburgh) extended this work to seven participants with profound motor deficits (Fugl-Meyer Assessment scores 15–35), implanted for four weeks; stimulation produced an average immediate improvement of 32% in strength and 5.6 Fugl-Meyer points, spasticity decreased in all participants, and cumulative gains of 6.6 Fugl-Meyer points were observed at study end despite only 8.6 h of total motor activity, suggesting a restorative as well as assistive effect analogous to the EESREHAB findings in SCI (Section 3.1) [71]. Because this application uses the same cervical epidural approach, similar afferent-recruitment mechanism, and even overlapping investigator groups as cervical ESCS for SCI upper-limb recovery (Section 3.2), post-stroke hemiparesis represents the non-SCI indication most directly informative for, and informed by, the SCI literature reviewed in this article.

### 10.2. Cardiac Autonomic Modulation: Heart Failure and Refractory Angina

The autonomic rationale developed for SCI cardiovascular applications in Section 4.1—using thoracic epidural stimulation to modulate sympathetic outflow—has a direct, and considerably less successful, analogue in heart failure. A first-in-man pilot trial of continuous high-thoracic (T1–T3) ESCS in patients with New York Heart Association class III systolic heart failure reported improvements in NYHA classification, quality of life, left ventricular end-systolic volume, and peak oxygen consumption [93]. However, this signal was not confirmed in the subsequent, larger DEFEAT-HF randomized trial, a 24-center study of 66 randomized and implanted patients, which found no significant difference between ESCS-on and ESCS-off arms in left ventricular end-systolic volume index or any secondary clinical endpoint, alongside a 16.7% rate of system- or procedure-related complications [72]. This divergence between an encouraging pilot study and a null, adequately randomized follow-up trial is instructive: it is the closest existing precedent for the kind of rigorous, controlled evaluation that the SCI literature (Section 7) still largely lacks, and it illustrates a genuine risk—that open-label, pilot-stage enthusiasm for a physiologically plausible mechanism may not survive controlled testing.

A related cardiac application, briefly noted here for completeness, is refractory angina pectoris, in which high-thoracic ESCS modulates sympathetic/parasympathetic balance to improve myocardial blood flow; a systematic review and meta-analysis of randomized controlled trials found that ESCS reduces anginal episode frequency and nitrate use in this population, giving this application a more established, if still pain-focused rather than SCI-analogous, evidence base than heart failure [94,95].

## 11. Conclusions

Epidural spinal cord stimulation, developed originally as a chronic pain therapy, has become a serious investigational tool for enabling, and in select cases durably improving, motor, cardiovascular, and pelvic-organ function after spinal cord injury. Evidence from the past four years—spanning activity-dependent spatiotemporal stimulation for walking, brain–spine interfaces, closed-loop cervical stimulation for hand function, and the recent refinement of thoracic, rather than lumbosacral, targeting for blood pressure normalization—has moved this field from isolated case reports to a broad, multi-institutional pipeline of feasibility and early-phase trials, summarized here by therapeutic domain (Table 1) and highest level of evidence (Table 2). Realizing the full clinical potential of ESCS for SCI will require larger, better-controlled (ideally sham-controlled) studies, validated responder biomarkers, dedicated safety monitoring during stimulation-parameter optimization, and continued device innovation—as well as the reimbursement pathways, training infrastructure, and expectation-calibrated communication called for through patient and clinician perspectives—but the trajectory of recent data supports cautious optimism that nontraditional ESCS indications will play an expanding role in SCI care over the coming decade. Parallel investigation in the two closely related non-SCI applications—most notably post-stroke hemiparesis—further supports the broader premise that epidural spinal stimulation is a maturing neuromodulation platform whose clinical reach is likely to continue expanding beyond its origins in pain management.

## Figures and Tables

**Table 1:** Selected ongoing and recently initiated trials of epidural spinal cord stimulation for nontraditional indications in spinal cord injury.

NCT/Registry ID	Title (Abbreviated)	Sponsor/Site	Primary Focus	Country/Region	Phase/Design	*N*	Status (October 2026)
**Motor/mobility (n = 19)**							
NCT07234903 [12]	ARC-IM Therapy to Support Recovery of Ambulatory Function in Subacute/Chronic SCI	CHUV/EPFL, Lausanne; Swiss Paraplegic Center, Nottwil	Lower-limb walking/ambulation	Switzerland	N/A; non-randomized, parallel, open-label	12	Recruiting
NCT07105878 [13]	Epidural Electrical Stimulation in Spinal Cord Injury	2nd Affiliated Hosp., Zhejiang Univ.	Motor recovery	China	N/A; single-arm, open-label	10	Recruiting (est. December 2026)
NCT07207798 [14]	Epidural Electrical Stimulation in SCI (T11–L3)	2nd Affiliated Hosp., Zhejiang Univ.	Motor recovery, level-specific	China	N/A; single-arm, open-label	10	Recruiting (est. December 2026)
ChiCTR2200061674 [15]	EMG-Induced Rehabilitation After Epidural Electrical Stimulation for SCI (RCT protocol)	Shanghai Ruijin Rehabilitation Hospital	EMG-induced rehabilitation after epidural stimulation; muscle strength (manual muscle testing)	China	N/A; randomized controlled, parallel, single-center	20	Pending (WHO ICTRP, Apr 2023)
ChiCTR2500099621 [16]	Functional Recovery After Epidural SCS in SCI (observational)	Academic (ChiCTR)	Observational functional recovery	China	N/A; observational, cross-sectional	30	Ongoing (study period to Feb 2027)
NCT05926843 [17]	SCS Assisted by Motor Rehabilitation Training for Restoring Motor Function After SCI	IRCCS Ospedale San Raffaele, Milan	Lower-limb motor recovery + locomotor training	Italy	N/A; single-arm, open-label	10 †	Completed
NCT06438991 [18]	Spinal Cord Stimulation for Lower Extremity Function	Mass General Brigham (Boston, MA)	Imaging biomarkers of stimulation responsiveness	USA	N/A; single-arm, open-label	10	Not yet recruiting
NCT05665998 [19]	Brain-Controlled SCS for Upper-Limb Rehabilitation (Cervical SCI)	CHUV, Lausanne (EPFL)	Closed-loop upper-limb recovery	Switzerland	N/A; single-arm, open-label	3	Recruiting
NCT06243952 [20]	Brain-Controlled SCS for Lower-Limb Rehabilitation	CHUV, Lausanne (J. Bloch, PI)	Closed-loop lower-limb recovery	Switzerland	N/A; single-arm, open-label	3	Ongoing (active, not recruiting)
NCT02936453 [21]	STIMO: EES With Robot-Assisted Rehabilitation in SCI	CHUV/EPFL, Lausanne	Foundational spatiotemporal stimulation + locomotor training	Switzerland	N/A; single-arm, open-label	12 †	Completed
NCT04632290 [22]	STIMO-BSI: Brain-Controlled Spinal Cord Stimulation in SCI	CHUV/EPFL, Lausanne	Brain–spine interface for naturalistic walking	Switzerland	N/A; single-arm, open-label	3	Ongoing (active, not recruiting)
NCT05644171 [23]	RESTORES: Restoration of Rehabilitative Function With Epidural Spinal Stimulation	National Neuroscience Institute, Singapore	Volitional motor control + overground walking, robotic neurorehab	Singapore	N/A; single-arm, open-label	18	Recruiting (to 2030)
NCT06225245 [24]	Augmenting Rehabilitation Outcomes and Functional Neuroplasticity Using Cervical Epidural Stimulation	Houston Methodist (Houston, TX)	Upper-limb neuroplasticity	USA	N/A; single-arm, open-label	12	Recruiting
NCT06455137 [25]	Epidural Electrical Stimulation for SCI Patients and Corticospinal Motor Circuit Improvement	Buddhist Tzu Chi General Hospital	Corticospinal circuit plasticity	Taiwan	N/A; single-arm, open-label	20	Recruiting
NCT06847295 [26]	Epidural Electrical Stimulation to Restore Standing and Walking in Chronic Paralysis	University of São Paulo General Hospital	Standing/walking, spasticity, QoL	Brazil	N/A; single-arm, open-label	10	Recruiting
NCT07306052 [27]	Epidural Spinal Cord Stimulation for Restoring Walking in SCI	2nd Affiliated Hosp., Zhejiang Univ.	Walking recovery	China	N/A; single-arm, open-label	12	Recruiting
NCT05966896 [28]	Epidural Stimulation to Restore Voluntary Movement Following SCI	University of Colorado Anschutz	Voluntary movement	USA	N/A; single-arm, open-label	2	Ongoing (enrolling by invitation)
NCT05095454 [29]	Short-Term Transcutaneous or Epidural Spinal Stimulation for Enabling Motor Function in SCI	Mayo Clinic	Comparative transcutaneous vs. epidural motor function	USA	N/A; randomized, parallel, open-label	6 †	Completed
NCT04736849 [30]	Epidural and Dorsal Root Stimulation in Humans With SCI	Mayo Clinic (Rochester, MN)	Mechanistic/afferent recruitment	USA	N/A; single-arm, open-label	22 †	Completed
**Cardiovascular/autonomic (n = 4)**							
NCT05111093 [31]	Epidural Electrical Stimulation to Restore Hemodynamic Stability and Trunk Control in SCI (HemON)	ONWARD Medical/CHUV	Cardiovascular regulation, trunk control	Switzerland	N/A; single-arm, open-label	20	Recruiting
NCT03924388 [32]	Spinal Cord Stimulation and Autonomic Response in People With SCI	Univ. of Calgary/Univ. of British Columbia	Autonomic/cardiovascular response	Canada	N/A; randomized, parallel, open-label	46	Unknown (last verified Dec 2019)
NCT03026816 [33]	E-STAND: Epidural Stimulation After Neurologic Damage	University of Minnesota	Volitional movement + cardiovascular function	USA	N/A; single-arm, open-label	100	Recruiting
NCT06410001 [34]	CE-STAND: Cervical Epidural Stimulation After Neurologic Damage	University of Minnesota (E-STAND consortium)	Cervical epidural stimulation, volitional movement + autonomic function	USA	Phase 1/2; single-arm, open-label	36	Recruiting
**Bladder, bowel, and sexual function (n = 3)**							
NCT05942339 [35]	BoxSwitch: ARC-IM Lumbar System to Support Mobility in Chronic SCI	CHUV/EPFL, Lausanne	Lower-limb mobility, at-home use, bladder/bowel/sexual function	Switzerland	N/A; single-arm, open-label	8	Recruiting
NCT06845904 [36]	Safety/Efficacy of Epidural Stimulation for Lower Urinary Tract Dysfunction After SCI	CHUV, Lausanne (J. Bloch, PI)	Bladder/lower urinary tract function	Switzerland	N/A; single-arm, open-label	3	Recruiting
NCT03452007 [37]	SPARC Bladder Mapping and Training Study	Academic (SPARC-affiliated)	Bladder mapping and training	USA	N/A; single-arm, open-label	10 †	Ongoing (active, not recruiting)
**Respiratory (n = 1)**							
NCT05178056 [38]	Spinal Cord Stimulation and Respiration After Injury	University of Louisville	Respiratory/diaphragmatic function	USA	N/A; randomized, parallel, single-blind	30	Recruiting

N = enrollment listed in the registry record (estimated target enrollment unless marked †, which denotes actual enrollment); N/A = no formal phase assigned in the registry record. Phase/design entries are taken from the registry record fields (phase, allocation, intervention model, and masking); values for ChiCTR2200061674 are from its published protocol [15] and the WHO ICTRP mirror record, and values for ChiCTR2500099621 are from the ChiCTR record. Sources: ClinicalTrials.gov and ChiCTR study records and associated news/institutional pages, accessed July–October 2026 (corresponding registry entries appear in the reference list). Recruitment status reflects the registry records as updated in October 2026. Readers should consult individual study/registry records for current enrollment status and phase, as registries are updated continuously and some ChiCTR-registered studies are not fully cross-indexed in English-language databases.

**Table 2:** Highest level of clinical evidence reported to date, by application.

Application	Highest Evidence Level Reported	Representative Study	Design
Lower-limb motor recovery (SCI)	Feasibility cohort, uncontrolled	Kathe et al., 2022 (EESREHAB) [46]	Single-arm, n = 9
Upper-limb/hand motor recovery (SCI)	First-in-human case study/preprint	BCI-ESCS study, 2025 [43]	Case study, n = 1 (preprint)
Cardiovascular regulation (SCI)	Within-participant controlled comparison	Phillips et al., 2025 [56]	Head-to-head crossover, n = 14
Bladder/bowel/sexual function (SCI)	Prospective cohort	Herrity et al., 2022 [65]	Prospective cohort, n = 7
Respiratory function (SCI)	Registered trial, not yet reported	NCT05178056 [38]	Early feasibility (ongoing)
Post-stroke upper-limb hemiparesis (non-SCI)	Feasibility trial, uncontrolled	de Freitas et al., 2026 [71]	Single-arm feasibility, n = 7
Cardiac autonomic modulation/heart failure (non-SCI)	Randomized controlled trial (null result)	Zipes et al., 2016 (DEFEAT-HF) [72]	RCT, n = 66

RCT = randomized controlled trial. “Feasibility/cohort” indicates open-label, uncontrolled studies (single-arm or small prospective cohorts) without randomization or sham control. Evidence level reflects the strongest design identified for that application in the literature reviewed here as of July 2026 and is not a formal GRADE or Oxford CEBM rating.

## Data Availability

No new data were created or analyzed in this study. All data discussed are available in the cited publications and in the ClinicalTrials.gov and ChiCTR registry records referenced in Table 1 and the reference list.
